# Retracted: Nomogram to Predict Overall and Cancer-Specific Survival in Patients with Synovial Sarcoma in the Extremities: A Population-Based Study

**DOI:** 10.1155/2023/9861979

**Published:** 2023-03-01

**Authors:** Computational Intelligence and Neuroscience

*Computational Intelligence and Neuroscience* has retracted the article titled “Nomogram to Predict Overall and Cancer-Specific Survival in Patients with Synovial Sarcoma in the Extremities: A Population-Based Study” [1] due to concerns that the peer review process has been compromised.

Following an investigation conducted by the Hindawi Research Integrity team [2], significant concerns were identified with the peer reviewers assigned to this article; the investigation has concluded that the peer review process was compromised. We therefore can no longer trust the peer review process, and the article is being retracted with the agreement of the Chief Editor.

The authors do not agree with the retraction.

## References

[1] Yang X.-Y., He X., Zhao Y. (2022). Nomogram to Predict Overall and Cancer-Specific Survival in Patients with Synovial Sarcoma in the Extremities: A Population-Based Study. *Computational Intelligence and Neuroscience*.

[2] Ferguson L. (2022). Advancing Research Integrity Collaboratively and with Vigour. https://www.hindawi.com/post/advancing-research-integrity-collaboratively-and-vigour/.

